# Bone marrow mesenchymal stem cells from leukemia patients inhibit growth and apoptosis in serum-deprived K562 cells

**DOI:** 10.1186/1756-9966-28-141

**Published:** 2009-11-03

**Authors:** Zhaohui Wei, Naiyao Chen, Hongxing Guo, Xueming Wang, Fangyun Xu, Qian Ren, ShiHong Lu, Bin Liu, Lei Zhang, Hui Zhao

**Affiliations:** 1 The Affiliated Hospital, North China Coal Medical college, Tanshan, Heibei, 063000, PR China; 2 State Key Laboratory of Experimental Hematology, Institute of Hematology, Chinese Academy of Medical Science & Peking Union Medical College, Tianjin 300020, PR China

## Abstract

**Background:**

The regulation of growth and apoptosis in K562 cells by human bone marrow mesenchymal stem cells (MSCs) from leukemia patients was investigated.

**Methods:**

K562 cells were cocultured with leukemic MSCs under serum deprivation. Cell Counting Kit-8 (CCK-8), PI staining, Annexin V/PI binding and FACS assays were used to investigate cell proliferation, cell cycle status, and apoptosis of K562 cells cultures in the presence or absence of 10% serum. Western blotting was used to determine the levels of Akt, phosphorylated Akt (p-Akt), the BCL-2 family member Bad, and phosphorylated Bad (p-Bad) proteins in K562 cells after coculturing with MSCs. The effects of LY294002 (a specific inhibitor of PI3K) on protein expression were also determined.

**Results:**

K562 cell proliferation was inhibited by coculture with MSCs and the dominant cell cycle was the G_0_-G_1 _phase. The proportion of apoptotic K562 cells was decreased and the levels of p-Akt and p-Bad were upregulated after exposing K562 cells to MSCs. However, when LY294002 was used, p-Akt and p-Bad proteins inK562 cells showed a significant reduction, while no distinct variation was seen in the nonphosphorylated Akt and Bad protein levels.

**Conclusion:**

Leukemic MSCs can inhibit K562 cell expansion and modulate the cell cycle to a state of relative quiescence. This allows the K562 cells to endure adverse conditions such as serum starvation. The PI3K-Akt-Bad signaling pathway may be involved in this antiapoptotic process via phosphorylation of the Akt and Bad proteins. Blocking MSC-induced transduction of the PI3K-Akt-Bad pathway may be a potential strategy for a targeted therapy to combat leukemia.

## Introduction

Bone marrow is not only the source of leukemic cells, but is also the primary site of leukemia relapse [1]. For these reasons, the hematopoietic microenvironment (HM) of the bone marrow plays a crucial role in the development and progression of leukemia. Variations in the HM may influence the biological behaviors of leukemia cells; for example, induction of resistance to chemotherapy drugs by hypoxia [2] is now known to involve many components. One important HM component is the bone marrow mesenchymal stem cell (MSC), which is involved in stabilizing microenvironments, and forming niches for cancer cells to endure adverse conditions.

Investigators have demonstrated normal MSCs and established MSC cell lines can protect leukemia cells from apoptosis [3-5]. However, the role of leukemic MSCs in the pathogenesis and prognosis of leukemia are still not well elucidated. What is known is that a substantial number of MSCs from leukemia patients are likely to differentiate into malignant cells and it is these cells that play multiple roles in directly regulating leukemia cells. However, the possibility that MSCs from patients with leukemia possess similar ability to modulate leukemia cells has not been well explored. Leukemic MSCs in all probability will aid in cell survival under adverse conditions (e.g., hypoxia, chemotherapy, serum deprivation). For this reason, we have designed a system that mimics a serum deprivation condition (i.e., fetal bovine serum (FBS) starvation) in order to observe the status of K562 cells and the influence of leukemic MSCs upon them.

The PI3K-Akt signal pathway and its downstream target BCL-2 family members play important roles in the induction and regulation of cell apoptosis, survival, proliferation and formation of the cellular framework [6]. Many studies have shown that activation of this signaling pathway in some leukemia cells continues for an extended duration [7-9]. An uncertain relationship still exists between the PI3K-Akt pathway and MSCs. Hence, the aim of the present study was to provide a preliminary outline of the variations of key proteins involved in the PI3K-AKt signaling pathway in leukemia cells.

## Materials and methods

### Cell line

Human chronic myelogenous leukemia cell line K562 was maintained in RPMI 1640 media supplemented with 10% fetal bovine serum (FBS), 100 U/ml penicillin, 100 U/ml streptomycin and 0.2 mmol/L glutamine at 37°C in a humidified incubator with a 5% CO_2 _atmosphere. Prior to the experiments, the K562 cells were suspended in complete DF-12 medium (Gibco, containing 10% FBS, 100 U/ml penicillin, 100 U/ml streptomycin and 0.2 mmol/L glutamine) or in DF-12 medium without serum.

### Isolation and characterization of human leukemic mesenchymal stem cells (MSCs)

Heparinized bone marrow from each patient (4 patients: 2 with chronic myelogenous leukemia in blast crisis, 1 with acute myelogenous leukemia, and 1 with acute lymphoblastic leukemia) was obtained after informed consent. The marrow was diluted twice with phosphate buffered saline (PBS), then isolated by Ficoll-Hypaque (Institute of Hematology) density-gradient centrifugation. Monocytes were collected by adherence to a plastic flask and incubated for 48 hrs in MSC conditioned medium containing 10% FBS, 0.2 mmol/L glutamine, 10-9 M Dex, 10 ng/ml EGF, 100 U/ml penicillin and 100 U/ml streptomycin. Medium was replaced at least twice a week and nonadherent cells were discarded. After 3-5 passages, the cells met the minimal criteria for defining multipotent mesenchymal stromal cells [10] with typical CD34-, CD14-, HLA-DR-, CD73+, CD90+, and CD105+, as identified by flow cytometry (data not shown). Fluorescein isothiocyanate labeled antibodies for the MSC immunophenotype were purchased from BD Pharmingen, except for CD105 antibody, which was phycoerythrin-labeled and purchased from Serotec. When MSCs were 80%-90% confluent, they were digested with trypsin and resuspended with MSC conditioning medium (supplemented with or without 10% serum) in preparation for experiments.

### Coculturing modifications for observing proliferation of K562 cells

#### Simple culture group (SCG group)

This group was divided into two subgroups based on culture media used. The SCG-N group represented the K562 cells cultured in completed DF-12 medium containing 10% FBS. The SCG-S group represented the K562 cells in DF-12 medium without serum. Both subgroups were cultured at 37°C in a humidified incubator with a 5% CO_2 _atmosphere for 72 hrs.

#### Contact culture group (CCG group)

MSCs were seeded into 24-well plates (Costar, Bodenheim, Germany) at the initial density of 1 × 10^4 ^cells/well, or 1 × 10^5 ^cells/well in 6-well plates (Costar, Bodenheim, Germany), and maintained in a 5% CO_2_, humidified atmosphere at 37°C for 24 hrs. The cells were then given a total gamma-irradiation of 15 Gy. Subsequently, K562 were seeded at 10^5 ^cells/well and cocultured with MSCs in 24-well plates for 24, 48 or 72 hrs. The K562:MSC ratio was 10:1, was selected according to previous literature[11]. The medium was supplemented with (CCG-N group) or without (CCG-S group) 10% FBS.

#### Separately cocultured group (Transwell group)

MSCs (1 × 10^4 ^initial cell count) were cultured for 24 hrs in the upper side of a transwell (NUNC Company, Denmark) chamber partitioned by a polycarbonate membrane (8.0 μm pore size, Corning Incorporated, Costar). These MSCs were then given a total irradiation of 15 Gy. After discarding the supernatant, the MSCs were cocultured with 1 × 10^5 ^of K562 cells in the lower part in DF-12 medium (with or without 10% FBS) at 37°C, 5% CO_2 _for 72 hrs.

#### Preparation for the conditioned medium group

MSCs were cultured in complete DF-12 medium at 37°C, 5% CO_2 _for 72 hrs, then the culture medium was harvested and centrifuged at 2,000 rpm for 10 min and stored at -80°C. This medium was doubled diluted with DF-12 medium without FBS then used to culture K562 cells for 72 hrs. The CM group included two subgroups cultured in conditioned medium with or without FBS.

### CCK-8 assay for detecting proliferation of K562 cells

Cells from the SCG, CCG, Transwell, and CM groups were cultured in DF-12 media with or without FBS for further observation. When cells were cocultured in different media for 72 hrs, cell proliferation was measured with a Cell Counting Kit-8 (Dojindo, Shanghai), following the manufacturer's instructions.

### Propidium iodide (PI) flow cytometric assay for determining cell cycle status under different nutritional states

The PI staining method was used for detecting the cell cycle status of cells of the SCG-N, CCG-N and CCG-S groups, using the manufacturer's protocol. Briefly, DNA was stained with 50 μg/ml propidium iodide (Sigma). Samples were kept for 1 hr in the dark at room temperature and the DNA index was then measured by cytofluorimetric analysis using an FACS Calibur flow cytometer (Becton Dickinson, San Diego, CA). Data were analyzed using CellQuest software.

### Annexin V/PI for cell apoptotic analysis

Cell viability was detected by trypan blue and apoptosis was evaluated by the annexin V/propidium iodide (BD Biosciences) double staining assay following the manufacturer's instructions. K562 cells were harvested at the end of treatment, rinsed twice with PBS, and stained with Annexin V-FITC apoptosis detection kit I (BD Biosciences). Analysis was performed on the FACS Calibur using CellQuest software.

### Western blotting

Three groups of K562 cells were cultured at 37°C, 5% CO_2 _for 24 hrs. SCG-S represented the group of K562 cells cultured without FBS. CCG-S represented the group of K562 and MSCs without FBS. CCG-S+LY294002 represented the group pretreated with 10 μM LY294002 for 1 hr. After incubation, K562 cells were dissolved in lysis buffer (100 mM Tris-HCl, pH 6.8, 4% SDS, 20% glycerin, 200 mM dithiothreitol, plus protease inhibitors) and quantified for proteins by a BCA protein assay kit (Pierce Company, USA). Equal amounts of protein extract were loaded onto a 12% SDS-PAGE gel and transferred to PVDF membrane (Gibaino Company, Beijing, China). The blot was blocked in 5% fat-free milk at 4°C overnight and then incubated with mouse monoclonal anti-Akt, p-Akt-Ser-473, anti-Bad, p-Bad-Ser-136 antibodies, (Cell Signal Transduction). Mouse monoclonal anti-beta-actin antibody (Cell Signal Transduction) was used as control. The immunocomplexes were visualized by using a chemiluminescent kit (Cell Signal Transduction).

### Statistical analysis

Data were presented as mean ± SD, using the SPSS system package for statistical analysis. Student-t-test was used for comparison of two groups of data. One-Way ANOVA was used for more than two groups of data. Multiple comparisons between two groups were analyzed by a SNK-q test. A P value < 0.05 was considered significant.

## Results

### MSCs inhibit proliferation of K562 cells under different nutritional conditions

**Figure 1:**
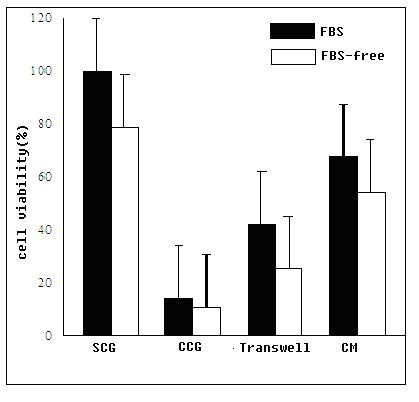
**MSCs inhibit the proliferation and viability of K562 cells**. Either in the present of MSCs or conditioned medium from MSCs, the suppression persisted signnificantly.

As shown in figure 1, the growth of K562 cells was clearly decreased in the absence of serum in culture media. However, even with the addition of 10% FBS, viable cell numbers in the coculture, transwell, and CM experimental groups were significantly decreased compared to the SCG subgroups (p < 0.001). The CCG groups were especially affected. This suggested that cell growth was inhibited when K562 cells were cocultured with MSCs Moreover, the suppression persisted even if the cells were separated in a transwell system or were cocultured in MSC supernatant, which indicated the suppression effect was mediated by some soluble substances, most likely cytokines.

### Effects of MSCs on K562 cell cycles

**Figure 2:**
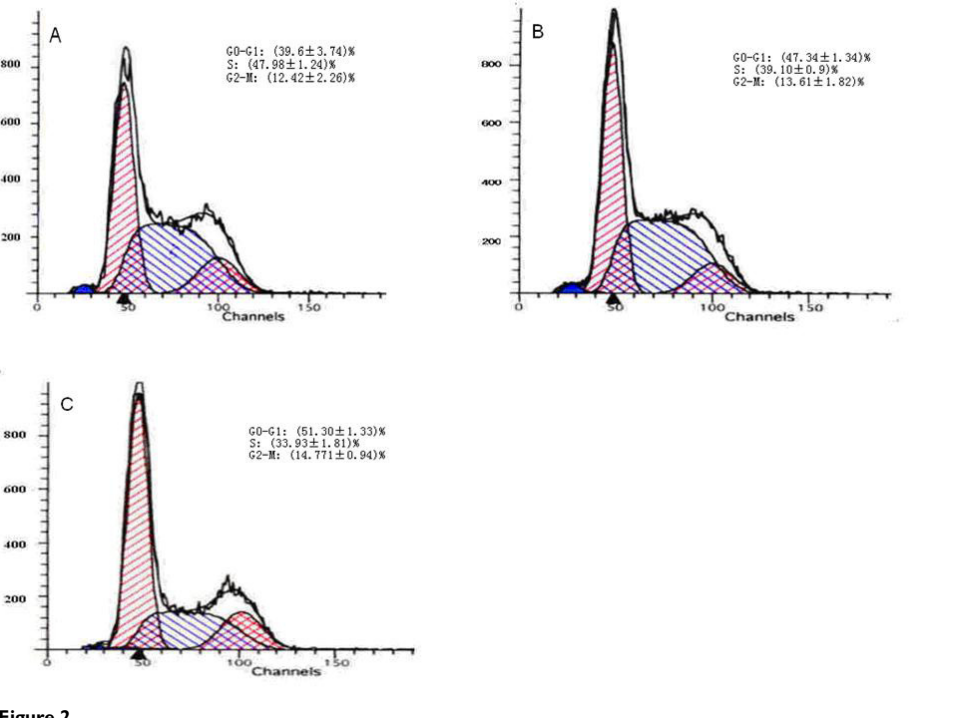
**Cell cycle distribution of K562 cells in SCG-N (A), CCG-N (B) and CCG-S (C) groups**. K562 cells were arrested in the G_0_-G_1 _phase by the presence of MSCs.

As shown in figure. 2, when compared with SCG-N group, the percentage of K562 cells in G_0_/G_1 _phase in the CCG-N group was dramatically increased, with a concomitant decrease in cells in the S phase. Moreover, with deficient nutrition, the CCG-S group showed further increases in the G_0_/G_1 _phase (39.60% vs. 51.30%) and reduction in the S phase (47.98% vs. 33.93%). Although there may have been an increased trend towards the G_2_-M phase, no significance difference was observed among the three groups. The presence of MSCs therefore reduced the numbers of leukemic cells in the S phase and increased the number of cells in the G_0_-G_1 _phase. K562 cells were arrested in the G_0_-G_1 _phase by the presence of MSCs. This pattern was more obvious under serum deprivation (p = 0.007).

### Effects of MSCs on the apoptosis of K562 cells

**Figure 3:**
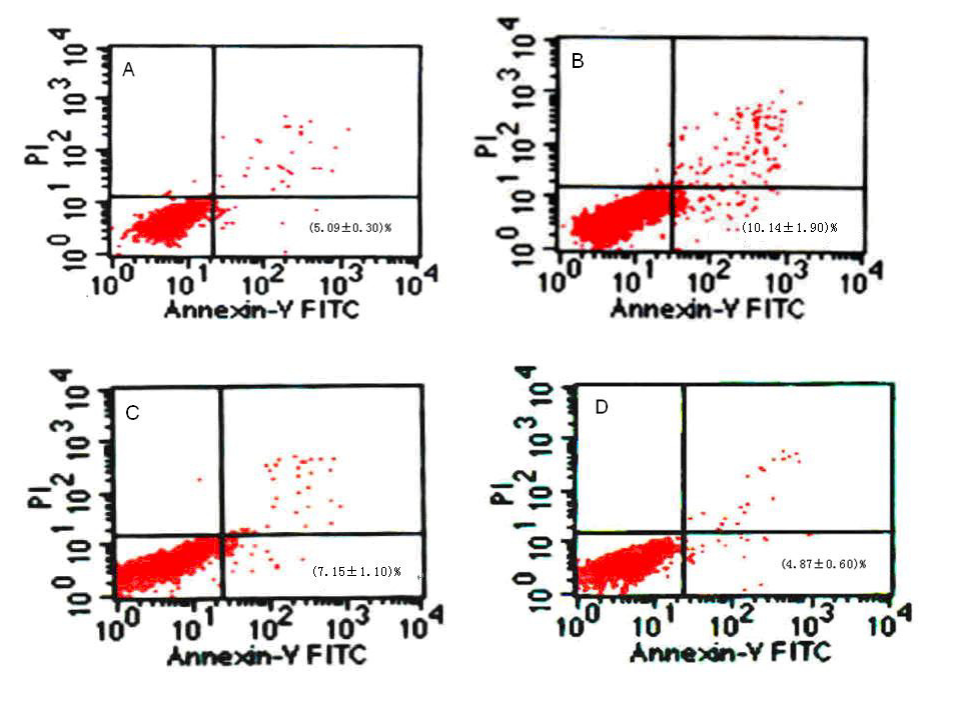
**Apoptotic percentages of K562 cells cultivated in different media**. (A), SCG-N, K562 cells cultivated in DF-12 with 10%FBS. (B), SCG-S, K562 cells cultivated without FBS. (C), K562 cells in CCG-S+MSCs+LY294002 group were pretreated with 10 μM LY294002 for 1 hr then cocultured with MSCs in DF-12 media without FBS. (D), CCG-S+MSCs, K562 cells cultivated with MSCs in the present of FBS-free medium.

The Annexin V/PI assay was used to detect apoptosis in K562 cells. As shown in figure 3, following FBS starvation for 24 hrs, the proportion of apoptotic K562 cells was significantly increased compared to that in groups supplemented with 10% FBS. After coculturing with MSCs, cell apoptosis was significantly decreased compared with SCG-S (p = 0.011), yielding results similar to those of the SCG-N group. However, in the presence of LY294002, the magnitude of the decrease in apoptosis was reduced (5.09% vs. 7.15%). As LY294002 is a the specific inhibitor of PI3K, the antiapoptotic ability of MSCs might have some relationship with the P13K signal pathway. Thus, we next examined the levels of known antiapoptotic proteins in K562 cells.

### Effects of MSCs on protein expression in K562 cells

**Figure 4:**
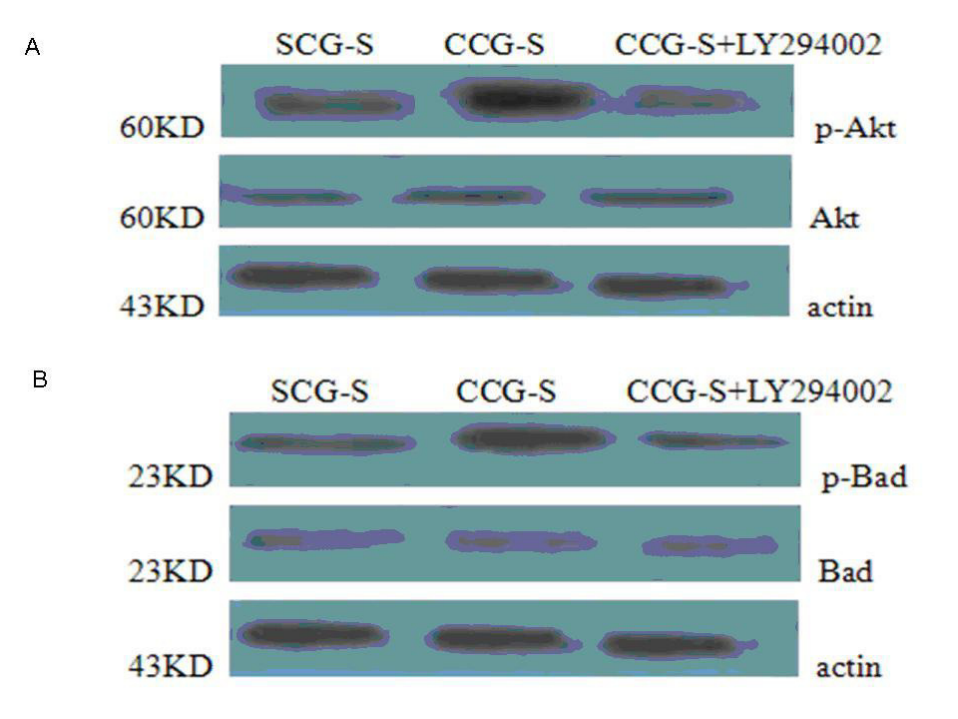
**Proteins of K562 cells identified by Western blot**. (A), Expressin of Akt, p-Akt proteins of K562 cells in SCG-S, CCG-S+MSCs and CG-S+MSCs+LY294002 groups. (B), Expressin of Bad, p-Bad proteins of K562 cells in SCG-S, CG-S+MSCs, CCG-S+MSCs+LY294002 groups. proteins were analyzed by Western blots with beta-actin as equally loading control (bottom). Independent experiments were repeated up to three times with the similar results.

Western blotting showed that the presence of MSCs raised the levels of the PI3K-Akt-related antiapoptotic proteins, p-Akt and p-Bad, in K562 cells. As shown in figure 4A, the 60KD band of Akt showed no significant difference among the SCG-S, CCG-S, CCG-S+LY294002 groups. In contrast, for the phosphorylated form p-Akt, expression levels were clearly higher in CCG-S group. Addition of LY294002 resulted in a reversal, with p-Akt level being similiar to that of the SCG-S group. These data indicate that the phosphorylation of Akt is apparently involved in the antiapoptotic process mediated by MSCs.

As shown in figure 4B, a band at 23 KD, representing the Bad and p-Bad proteins in K562 cells, also showed obvious increases in the phosphorylated form of Bad in the CCG-S group. Upregulation was nearly reversed by treatment with LY294002, which causes an upstream blockade of PI3K. There were no significant variations among the Bad levels of these groups.

## Discussion

As evidence on bone marrow HM has accumulated over the past few years, it has become widely acknowledged that MSCs affect a great number of different cell types besides hematopoietic parenchymal cells, including leukemia cells [11-13]. With this close relationship between MSCs and leukemia cells, it may be that the influence of MSCs is what ultimately determines the prognosis of leukemia.

In general, MSCs in the HM have been considered to be nurse-like cells that exert a form of protective modulation. Leukemic MSCs can reportedly inhibit the chemotherapeutic-induced apoptosis of Jurkat cells and HL-60 cells. Moreover, they can interfere with the cell cycle of Jurkat cells at the G0-G1 phase [14,15]. They can also negatively regulate cancer immunotherapy involving NK cells and inhibit cytotoxic T cells by secreting cytokines [16,17]. Thus, there appear to be multiple roles of MSCs in proliferation, differentiation, and survival of leukemia cells [18-20] as well as normal immune cells. In the present study, the role of leukemic MSCs on K562 cells was explored under normal nutritional conditions or under serum starvation. We noticed a marked increase in K562 cell apoptosis after serum starvation for 24 hours. However, a marked decrease in apoptosis was observed when these starved cells were cocultured with MSCs, supporting the protective role of leukemic MSCs against apoptosis. This inhibition existed both in contact coculture and in separated coculture, and was induced even by supernatant culture medium from MSCs. Thus, our data support that cytokines, adherent reactions and gap junctions participated in inhibiting leukemic cell proliferation.

When K562 cells were cocultured with normal MSCs, they also showed cell cycle blockade. These K562 cells also showed drug-resistance to daunorubicin (DNR), which is consistent with their increased G_0_-G_1 _phase and reduced S phase. The reasons for this drug resistance may also relate to the upregulation of antiapoptotic gene expression and the cytokines secreted by MSCs. Our data also showed a similar cell cycle blockade of K562 cells resulting from coculture with leukemic MSCs obtained from 4 patients, although these nurse cells may have undergone malignant transformation in vivo or over their long time in culture. Inhibition of cell growth is a primary method of treating leukemia; however, the blockade of the cell cycle may prevent the efficacy of chemotherapeutic agents, which mainly target the proliferative phase of tumor cells. When most tumor cells are blocked at the quiescent phase, they may evade the killing powers of chemotherapeutics and may ultimately form micro residual disease (MRD). We hypothesize that leukemic MSCs may provide a niche for tumor stem cells, in which K562 cells back up the proliferation and self-renewal potential. These tumor cells may then be the source of relapse.

Constitutive activation of Akt, one downstream target of PI3K, is also believed to promote proliferation and increase cell survival, leading to cancer progression[21]. The PI3K-Akt signal pathway is involved in the antiapoptotic activity of tumor cells and culminates in the phosphorylation of the BCL-2 family member, Bad, thereby suppressing apoptosis and promoting cell survival. Akt phosphorylates Bad both in vitro and in vivo, and blocks Bad-induced cell death [22]. The PI3K-Akt-Bad pathway may represent a form of general antiapoptotic machinery, although there is insufficient evidence to support this hypothesis at present.

We determined the expression levels of Akt, p-Akt, Bad, p-Bad proteins in K562 cells after inoculation with MSCs. Under the condition of K562 cells alone, there was a basal expression of p-Akt, and p-Bad, which might have been related to the bcr/abl fusion protein-activated PI3K-Akt signal pathway. In addition, the expression of p-Akt and p-Bad was increased after coculture with leukemic MSCs. The addition of the specific inhibitor LY294002, which competes with PI3K for ATP binding sites [23], resulted in a dramatic decrease in levels of both phosphorylated proteins, while no obvious difference in Akt and Bad expression was observed among the three groups. Hence, we showed that the PI3K-Akt pathway was activated after coculture with MSCs. The pro-apoptotic molecule, Bad, was then phosphorylated and exerted inhibitory effects on starvation-induced apoptosis.

Taken together, serum deprivation appears to mimic the effects of an adverse HM for leukemia cells. MSCs of leukemia patients can retard the cell cycles of K562 cells, inhibiting their proliferation and reducing their apoptosis. Consequently, MSCs protect leukemia cells against adverse conditions like serum deprivation and ultimately sustain their viability. The activation of the PI3K-Akt-Bad signaling pathway seems to be involved in the protective machinery. Therefore, approaches that block the activation of this signaling pathway may in turn remove this shielding and consequently may prove to be of benefit in the effective treatment of leukemia.

## Competing interests

The authors declare that they have no competing interests.

## Authors' contributions

ZHWcontributed conception, designed the study carriedouttheexperiments, collected and interpretated the data, and wrote the manuscript. NYC ributed conception, designed the study and wrote the manuscript. HXG carriedouttheexperiments, collected and interpretated the data. XMW carriedouttheexperiments, collect ed and interpretated the data. FYX assisted with study implementation. QR and SHL assisted with study implementation and supervised laboratory procedures. BL carriedouttheexperiments, collected and interpretated the data. LZ supervised laboratory procedures. HZ contributed conception, analyzed the data, and wrote the manuscript. Allauthorsreadandapprovedthefinalmanuscript.
